# Animal models of Parkinson’s disease: bridging the gap between disease hallmarks and research questions

**DOI:** 10.1186/s40035-023-00368-8

**Published:** 2023-07-19

**Authors:** Axelle Dovonou, Cyril Bolduc, Victoria Soto Linan, Charles Gora, Modesto R. Peralta III, Martin Lévesque

**Affiliations:** 1 CERVO Brain Research Centre, 2601, Chemin de la Canardière, Québec, QC G1J 2G3 Canada; 2 Department of Psychiatry and Neurosciences, Faculty of Medicine, Université Laval, Québec, QC Canada

**Keywords:** Parkinson’s disease, Animal model, Alpha-synuclein, Transgenic model, Preformed fibril, Mouse

## Abstract

Parkinson’s disease (PD) is a progressive neurodegenerative disorder characterized by motor and non-motor symptoms. More than 200 years after its first clinical description, PD remains a serious affliction that affects a growing proportion of the population. Prevailing treatments only alleviate symptoms; there is still neither a cure that targets the neurodegenerative processes nor therapies that modify the course of the disease. Over the past decades, several animal models have been developed to study PD. Although no model precisely recapitulates the pathology, they still provide valuable information that contributes to our understanding of the disease and the limitations of our treatment options. This review comprehensively summarizes the different animal models available for Parkinson’s research, with a focus on those induced by drugs, neurotoxins, pesticides, genetic alterations, α-synuclein inoculation, and viral vector injections. We highlight their characteristics and ability to reproduce PD-like phenotypes. It is essential to realize that the strengths and weaknesses of each model and the induction technique at our disposal are determined by the research question being asked. Our review, therefore, seeks to better aid researchers by ensuring a concrete discernment of classical and novel animal models in PD research.

## Introduction

**Fig. 1:**
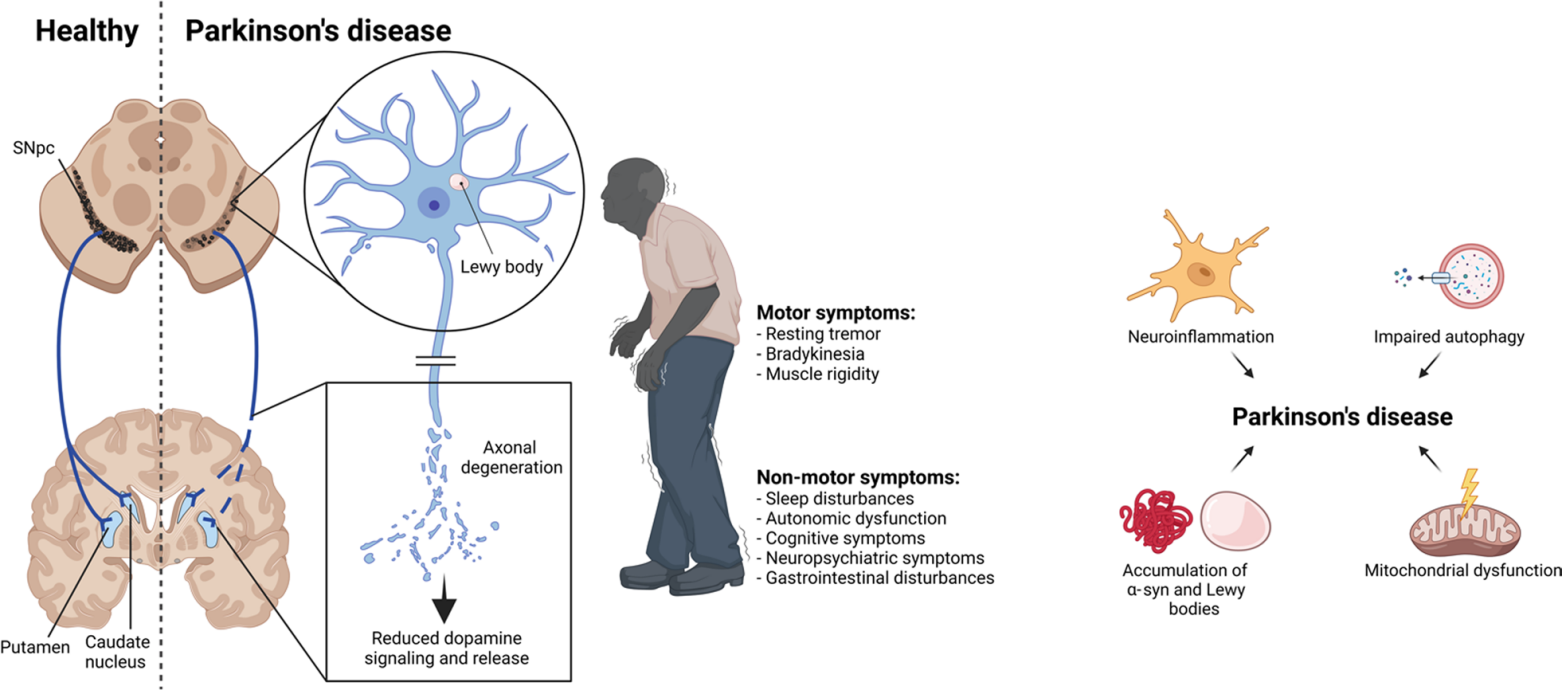
The hallmarks of Parkinson’s disease (PD). Predominant PD motor symptoms arise from DA neuron loss in the SNpc, and the denervation of their axons in the caudate nucleus and putamen, also called the striatum. The cause of PD remains unclear but involves loss of DA neurons, α-syn aggregation, mitochondrial dysfunction, autophagy impairments, and neuroinflammation. PD patients additionally exhibit non-motor symptoms. The figure was generated using BioRender.

Parkinson’s disease (PD) is the second most common age-related neurodegenerative disease, affecting up to 3% of individuals aged 65 and older [1, 2]. The disease is a progressive, multifactorial, and heterogeneous disorder. PD patients show motor dysfunction, such as resting tremors, rigidity, and bradykinesia [3, 4]. These cardinal symptoms are caused by the degeneration of dopamine (DA) neurons in the substantia nigra pars compacta (SNpc). PD is also accompanied by non-motor symptoms, including sleep disorders and dysfunction, autonomic dysfunction, cognitive and neuropsychiatric symptoms, gastrointestinal symptoms, weight and visual disturbances, and fatigue [2]. The pathology arises alongside the accumulation of Lewy bodies composed of aggregated α-synuclein (α-syn). The cause of DA neuronal loss remains unclear, but several pieces of evidence link these neuropathologic processes to impaired proteasomal protein clearance, mitochondrial dysfunction, and neuroinflammation [5, 6] (Fig. 1). Despite being well-characterized, the diagnosis of PD is only confirmed with a post-mortem autopsy, and what triggers this disease is still not clearly understood.

Animal models have only been able to reproduce partial signs of the PD pathology. Compared to the clinical characteristics seen in patients, the onset, progression, and disease outcome in these models remain incomplete. Nevertheless, these animal models are still necessary to study the complexity of the brain networks involved in brain pathologies or to evaluate the impact of potential therapeutic approaches. The choice of the animal model is crucial, and studies must be interpreted with the inherent limitations of the paradigm chosen. In this review, we encompass a wide range of animal species, including rodents, non-human primates, and non-mammalian organisms such as *Drosophila* and *C. elegans*. Our aim is to provide an updated and comprehensive overview of both conventional and unconventional animal models of PD, including models that have received comparatively less attention. Moreover, our review goes beyond the primary pathogenic hallmarks of PD and sheds light on the secondary traits observed in each model, such as mitochondrial and autophagic dysfunctions, as well as neuroinflammation. By offering researchers a valuable resource, our review aims to assist them in selecting the most relevant animal model that aligns with their specific scientific questions.

## Drug-induced parkinsonism

Drug-induced parkinsonism (DIP) is the most common form of secondary parkinsonism, meaning that in patients, DIP produces symptoms similar to those of PD. Typical symptoms in DIP include olfactory dysfunction, difficulty starting and controlling movement, loss or weakness of movement, and resting tremors [7]. DIP is the second most common cause of parkinsonism after idiopathic PD [8, 9]. In the following subsections, we will summarize existing drug-induced models, which reproduce several motor impairments in animals but fail to replicate the majority of neuropathologies associated with PD.

### Reserpine

**Fig. 2:**
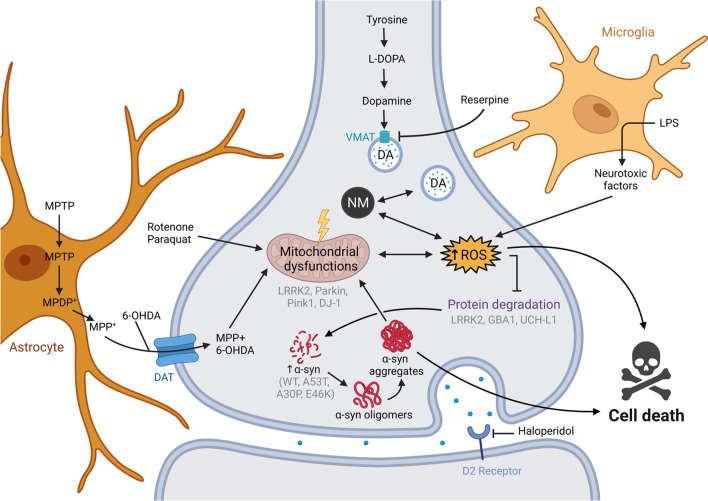
Schematic summary of the current known mechanisms that trigger DA neuron death, and the action of different genes and compounds used to model PD. The loss of DA neurons could result from impaired protein degradation, mitochondrial dysfunction, α-syn aggregation, and neuroinflammation, which can be induced by neurotoxic or genetic alteration. The reduced DA signaling through drug inhibition could also lead to a PD-like phenotype. LPS: lipopolysaccharide, DAT: dopamine transporter, VMAT: vesicular monoamine transporter, NM: neuromelanin, DA: dopamine. The figure was generated using BioRender.

One of the first animal models of PD was generated with the injection of reserpine in 1957 by Carlsson and colleagues [10]. This molecule is an inhibitor of the vesicular monoamine transporter (VMAT) type 2 (Fig. 2) [11]. Reserpine administration causes monoamine depletion in nerve terminals by reducing vesicular storage and release. This loss of monoamines leads to hypolocomotion and muscular rigidity. Firstly used as an antihypertensive drug due to its capacity to deplete cellular monoamine content [12], the clinical application of reserpine resulted in patients developing lethargy, depression, and motor dyskinesia [12–14]. In rodents, administration of reserpine induces motor impairment as well as memory, cognitive, and emotional deficits [15, 16]. Injection of reserpine partially mimics the pathogenesis of PD, developing akinesia and rigidity that reflect the clinical features of the disease. Furthermore, the work of Carlsson and colleagues showed that Levodopa (L-DOPA) can partially rescue the effect of reserpine administration [10]. In rats, reserpine administration produces sexually dimorphic impairments in motor performance, as present in PD [17]. Within weeks, DA neuronal loss in the SNpc and fibers in the dorsal striatum can be seen. However, this phenotype is transient. A partial recovery of nigral DA neurons occurs 30 days post-injection, rescuing motor deficits [16–18]. It should be noted that no significant loss of DA axonal innervation in the dorsal striatum is observed in female rats, which could explain the dimorphic impairments in motor behavior [17]. Overall, reserpine can induce PD symptoms in humans and parkinsonian-like signs in rodents (Table 1). The facility for adjusting dose concentration and administration allows multiple levels of studies of PD symptomatology. Nevertheless, the reserpine model is not commonly used to study late-stage hallmarks of PD pathology, because it fails to generate Lewy body-like inclusions [19] and permanent loss of DA neurons. While there are reports of oxidative stress in the striatum of rodents upon treatment with reserpine, there is neither indication of mitochondrial or lysosomal dysfunction, nor inflammation [20]. However, other than memory deficits, reserpine also generates several non-motor features relevant to the preclinical manifestations of PD, i.e., sleep abnormalities, anxiety, depressive-like behavior, and gastrointestinal dysfunction [21]. For this reason, reserpine can be used as a model to study the disease progression and neurochemical features of PD.

**Table 1:** Summary of the pathogenic characteristics observed in PD animal models

Model	Species	Dopamine loss	Motor deficits	α-syn pathology	Mitochondrial dysfunction	Autophagic impairment	Neuro- inflammation	Comments
*Drug-induced parkinsonism*								
Reserpine	Rodents	Yes (SNpc and Striatum)	Yes	Yes	NR	NR	NR	Oxidative stress Non-motor phenotype Transient loss of DA neurons Sexual dimorphic effect
Haloperidol	Rodents, primates	Yes (Striatum)	Yes	No	Yes	NR	Yes	Crosses the BBB Induces features of neuroinflammation No loss of SNpc DA neurons
*Neurotoxic animal models*								
6-OHDA	C. elegans*, rodents, primates	Yes (SNpc and Striatum)	Yes	No	Yes	Yes	Yes	Oxidative stress Non-motor phenotype Does not cross the BBB
MPTP	Rodents and primates	Yes (SNpc and Striatum)	Yes	Yes	Yes	Yes	Yes	Oxidative stress Recreates phases of PD Non-motor phenotype Phenotype variability in rodents
LPS	Rodents	Yes (SNpc and Striatum)	Yes	Yes	Yes	Yes	Yes	Oxidative stress Non-motor phenotype No α-syn inclusions Tau pathology
*Agrochemical-induced animal models*								
Rotenone	C. elegans*, Drosophila* and rodents	Yes (SNpc and Striatum)	Yes	Yes	Yes	Yes	Yes	Oxidative stress Non-motor phenotype Inter-individual variability
Paraquat & Maneb	C. elegans*, Drosophila* and rodents	Yes (SNpc)	Yes	Yes	Yes	NR	Yes	Combination increases toxicity Oxidative stress Non-motor phenotype Intact striatal fibers in rats Induces pulmonary fibrosis
*Transgenic models*								
PRKN & PINK1	C. elegans* (KO)	Yes	Yes	N/A	Yes	NR	NR	Lifespan decreases
	Drosophila* (KO)	Yes	Yes	N/A	Yes	NR	NR	Degeneration of flight muscles No DA cell loss in *PRKN* ortholog-KO *Drosophila*
	Mice (KO)	Yes (SNpc and striatum)	Yes	No	Yes	No	Yes	Mitochondrial dysfunction Slow degeneration
	Primates (KO)	Yes (SNpc)	Yes	NR	NR	NR	NR	Years to develop the phenotype
LRRK2	C. elegans* (OE)	Yes	Yes	N/A	Yes	Yes	NR	Progressive loss of DA neurons
	Drosophila* (OE)	Yes	Yes	N/A	Yes	Yes	NR	Synaptic alterations Tau pathology
	Mice (OE)	Yes (SNpc and Striatum)	Yes	Yes	Yes	Yes	Yes	Oxidative stress Synaptic alterations Tau pathology
DJ-1	Mice (KO)	Yes (SNpc and Striatum)	Yes	No	Yes	Yes	NR	May target early phases of pathology Age-dependent phenotype
UCH-L1	Drosophila* (KO)	Yes	Yes	N/A	NR	NR	NR	Progressive age-dependent loss of SNpc DA neurons
	Mice (OE)	Yes (SNpc and Striatum)	Yes	No	NR	NR	NR	Increased vulnerability to α-syn Not well characterized
GBA1	Drosophila* (KO/KI)	Yes	Yes	N/A	NR	NR	NR	Increases aggregation in mutant α-syn model
	Mice (KI)	Yes	Yes	Yes	Yes	Yes	Yes	Cognitive deficits No α-syn aggregates in *GBA1* L444P heterozygous mice
Lmx1a/b	Mice (KO)	Yes (SNpc and Striatum)	Yes	Yes	Yes	Yes	NR	Altered development KO needs to be performed on mature animals
Otx2	Mice (KO)	Yes (SNpc and Striatum)	Yes	N/A	N/A	N/A	N/A	Loss of DA neurons in the VTA Altered development KO needs to be performed on mature animals
Foxa1/2	Mice (KO)	Yes (SNpc and Striatum)	Yes	No	N/A	N/A	N/A	Altered development KO needs to be performed on mature animals
Pitx3	Mice (KO)	Yes (SNpc and Striatum)	Yes	N/A	N/A	N/A	N/A	Altered development KO needs to be performed on mature animals
Nurr1 & En1	Mice (KD)	Yes (SNpc and Striatum)	Yes	N/A	N/A	N/A	N/A	Total depletion is not viable (Altered development) KD needs to be performed on mature animals
c-Rel	Mice (KO)	Yes (SNpc and Striatum)	Yes	N/A	N/A	N/A	N/A	Altered development KO needs to be performed on mature animals
Mitochondrial impairment	Mice (POLG)	No	No	NR	Yes	NR	NR	No phenotypic accentuation in *DJ-1*/*PRKN* deficient mice
	Mice (*Ndufs2* KO)	Yes (Striatum)	Yes	NR	Yes	NR	NR	Total reduction of MCI activity Mouse death at 5–6 months
	Mice (*Ndufs4* KO)	Yes (Striatum)	No	Yes	Yes	NR	NR	Partial reduction of MCI activity No loss in SNpc
*α-syn models*								
WT α-syn	Mice (M61)	Yes (Striatum)	Yes	Yes	Yes	NR	Yes	No degeneration of SNpc DA neurons
	C. elegans* and Drosophila*	Yes	Yes	Yes	Yes	NR	NR	No endogenous α-syn
C-terminally truncated α-syn	Mice (MI2)	Yes (SNpc and Striatum)	Yes	Yes	NR	NR	NR	Null endogenous α-syn background α-syn expression restricted to DA neurons
E46K human α-syn	Mice (M47 line)	NR	Yes	Yes	NR	NR	Yes	No α-syn in SNpc DA neurons
A30P human α-syn	Mice	NR	Yes	Yes	Yes	Yes	NR	Around a year to develop strong phenotype No degeneration of SNpc DA neurons
	C. elegans* and Drosophila*	Yes	Yes	Yes	Yes	NR	NR	Lower α -syn accumulation than α-syn WT Lower α -syn accumulation than α-synA53T
A53T human α-syn	Mice (M83 line)	NR	Yes	Yes	Yes	Yes	Yes	α-syn inclusions in non-PD-related areas
	C. elegans* and Drosophila*	Yes	Yes	Yes	Yes	NR	NR	No endogenous α-syn
PFFs	Mice	Yes	Yes	Yes	NR	NR	Yes	Slow phenotype development in WT mice (6 months) Accelerated phenotype in A53T transgenic mice
	Primates	Yes	NR	Yes	NR	NR	NR	-
*Viral-vector-mediated models*								
α-synuclein viral expression	Rodents and primates	Yes (SNpc and Striatum)	Yes	Yes	Yes	Yes	Yes	Progressive neuronal loss Non-motor phenotype Variable phenotype depending on the injection Non-physiological α-syn expression
NM-like production	Rodents	Yes (SNpc and Striatum)	Yes	Yes	Yes	Yes	Yes	Age-dependent progression

* *C. elegans* and *Drosophila* do not express endogenous α-synuclein

BBB: blood-brain barrier; KO: Knock-Out; KI: Knock-In; N/A: Not applicable; NM: neuromelanin; NR: Not reported *in vivo;* OE: Overexpression; PFF: preformed fibril; POLG: polymerase gamma; SNpc: substantia nigra pars compacta

### Haloperidol

Haloperidol is a typical antipsychotic that binds post-synaptic dopaminergic D2 receptors [22] (Fig. 2). The block of striatal DA transmission results in abnormal downstream firing within the basal ganglia circuits, manifesting muscle rigidity and catalepsy [23, 24]. Acute administration of haloperidol also reduces the striatal content of DA, noradrenaline, and serotonin [25]. A prolonged administration in mice causes mitochondrial complex I (MCI) deficiency in the frontal cortex, hippocampus, striatum, and midbrain [26]. The haloperidol model is used for modelling rigidity, dyskinesia or catalepsy, and for discovering novel antiparkinsonian agents in rodents [27–30] and non-human primates [31–34]. Additionally, chronic treatment of rodents with haloperidol results in significant increases in the levels of pro-inflammatory cytokines TNF-α and IL-1β in the cortex and striatum compared to control animals [35]. However, no midbrain DA neuron degeneration has been reported with haloperidol. Thus, haloperidol may be less relevant for studying novel neuroprotective or neurorepair strategies for PD (Table 1).

## Neurotoxic models

### 6-Hydroxydopamine (6-OHDA)

Identified more than 60 years ago [36], the neurotoxin 6-OHDA is widely used to model PD by lesioning the nigrostriatal DA system [37–39]. 6-OHDA is a highly oxidizable DA analogue that transfers through the dopamine transporter (DAT) and the noradrenaline reuptake transporter. It exerts cytotoxic effects through different pathways: production of free radicals, and direct inhibition of MCI in the respiratory chain [40, 41] (Fig. 2). These different mechanisms may be linked to the generation of reactive oxygen species (ROS) and the release of cytochrome *c*, which leads to the activation of astrocytes and microglia [42, 43]. Because 6-OHDA does not cross the blood-brain barrier (BBB), an intracranial injection is required to exert its toxic effects [44]. In the past, at least three 6-OHDA lesional models have been used to mimic PD: an injection into the medial forebrain bundle, the SNpc, or the terminal regions of the nigrostriatal pathway in the striatum. The intravenous co-administration of desipramine helps protect the noradrenergic system from damage, and allows the targeting of catecholaminergic neurons, such as the DA neurons of the SNpc [40]. The 6-OHDA injection in the striatum leads to more progressive degeneration of DA neurons. In rodents, axonal denervation can be seen 3 h after injection in the striatum, while reduction of tyrosine hydroxylase (TH)-positive neurons in the SNpc is delayed by several days post-surgery [45, 46]. By comparison, an injection of the neurotoxin into the medial forebrain bundle or directly into the SNpc results in more severe and rapid neurodegeneration [47]. These injections result in motor impairments and non-motor phenotypes in rodents that include cognitive and gastrointestinal dysfunction [48–51]. This toxin not only induces a reduced expression of the lysosomal-associated membrane protein 1, but also impairs the hydrolase activities of lysosomal proteases [52]. 6-OHDA is not accompanied by α-syn aggregation or the formation of Lewy body-like inclusions. The 6-OHDA model is also used in *Caenorhabditis elegans* (*C. elegans*) [53], mainly for rapid studies such as drug screenings. More used in rodents, specifically in rats, the time course and severity of the 6-OHDA PD pattern depends on the amount and the injection location in the brain. No model has been developed yet for *Drosophila melanogaster* (*D. melanogaster*).

In summary, 6-OHDA is an affordable drug frequently used to generate rapid and specific neurodegeneration of the nigrostriatal system (Table 1). This model mimics several cellular processes identified in PD and is suitable for studying the molecular basis of cytotoxicity, oxidative stress processes, neuroinflammation, and neuronal death [43, 54, 55]. Although the 6-OHDA model has many advantages, its course differs from that observed in idiopathic PD, where neuronal degeneration occurs over the years. Despite its limitations, it remains an excellent tool for studying anti-PD drugs, *L*-DOPA-induced dyskinesia, or cell transplantation therapy.

### 1-Methyl-4-phenyl-1,2,3,6-tetrahydropyridine (MPTP)

First discovered in humans, this neurotoxin soon became a vital research tool in the early 1980s. Mistaken for synthetic heroin, MPTP produced clinical and pathological PD features in young drug addicts [56]. After systemic administration, MPTP rapidly crosses the BBB and is metabolized to MPDP+ (1-methyl-4-phenyl-2,3-dihydropyridinium) by astrocytes [57]. The active toxic compound, 1-methyl-4-phenylpyridinium (MPP+), quickly enters DA neurons through DAT, where it binds to the VMAT and translocates to synaptosomal vesicles or remains within the mitochondria and cytosol (Fig. 2). MPP + leads to an acute deficit in ATP formation and ROS production, via binding to MCI, and ends in neuronal death [58]. Furthermore, MPTP administration drives microglial activation and cytokine release [59]. In humans and non-human primates, MPTP can replicate almost all the motor impairments of PD, including tremors, rigidity, slowness of movement, postural instability, and freezing [60]. The loss of SNpc DA neurons can also be accompanied by an almost complete depletion in DA and associated metabolites in the putamen [61]. It must be noted that this model is highly variable depending on the neurotoxin dosage, and the species and strains used. Unlike primates, rodents (and variable strains of mice) are less sensitive to MPTP toxicity and require multiple doses over several days to induce neuronal degeneration. Thus, an appropriate administration schedule is needed [62]. While mice treated with MPTP do not develop persistent and progressive motor dysfunctions [60], they are arguably a viable choice for studying neuroanatomical and neurochemical alterations. In the SNpc and ventral tegmental area (VTA) of treated mice, MPTP causes the loss of more than half of all DA neurons. Recent studies show that the administration of MPTP can increase the total and the phosphorylated α-syn as well as tubulin-associated unit (Tau) in the hippocampus and SNpc [63]. Hence, MPTP is widely used as a model to study the molecular and neuropathological events in PD, especially in non-human primates (Table 1).

### Lipopolysaccharide (LPS)-induced neuroinflammation

In PD patients, a dense population of reactive microglia and astrocytes can be found in the brain, accompanied by an increased amount of pro-inflammatory cytokines, suggesting that neuroinflammation could play a key role in the pathogenesis [64]. Neuroinflammation can be modeled using LPS, an endotoxin produced by Gram-negative bacteria and is well-known for its pro-inflammatory properties. LPS binds the toll-like receptor 4 mainly expressed in microglia [65]. The use of LPS was initially intended for systemic inflammation models and the discovery of novel therapies in the treatment of acute inflammation [66, 67]. However, in 2001, a clinical case report showed that a 22-year-old laboratory worker developed PD syndrome after accidental exposure to *Salmonella minnesota* LPS through an open wound. Positron emission tomography confirmed that the subject also suffered from a dopaminergic neuronal loss in the SNpc [68]. Furthermore, two studies published earlier showed that injecting LPS into the SNpc of rats leads to neurodegeneration of nigral DA neurons [69, 70]. The neuronal loss is permanent and selective to DA neurons [71]. Since then, several LPS-induced models of PD have been developed, but their phenotypes greatly depend on the administration route [65, 72]. LPS can be delivered systemically or locally to the striatum, pallidum, or SNpc through intracerebral injections or intranasally.

While a few studies have used mice, LPS is more commonly used in rats [73–75]. Intrastriatal and intranigral injection of LPS in rats results in severe neurodegeneration of DA neurons from the SNpc with reduced striatal DA and motor impairments within a month [76–83]. Reduced MCI and II can be observed in nigral neurons [78]. A similar phenotype is seen when injecting LPS into the pallidum of rats, but motor impairments appear transient and return to normal after a few weeks [84]. It should be noted that using older rats can increase the severity of the phenotype [78, 84]. Furthermore, it has been reported that LPS increases the expression of α-syn in the brain [78, 85], the amount of oligomerized α-syn [86], and the expression of cytokines and activated microglia in both the SNpc and the striatum [78, 82]. Overall, local delivery of LPS to the brain generates features relevant to PD and can thus be used to clarify the role of neuroinflammation in SNpc DA neuron degeneration. However, the pathology is limited to the injection site.

Systemic injection of LPS in mice through intraperitoneal injection induces loss of DA neurons, motor deficits, and α-syn accumulation [85, 87, 88]. Autophagic impairment has also been described in nigral neurons with this route of administration [89]. It has been reported that there may be a delay in the appearance of the phenotype depending on the recurrence of the dose injected [72]. After a single intraperitoneal injection of LPS in mice, neurodegeneration of DA neurons can take several months [87, 88]; this delay can be reduced to 1 week by performing daily injections of LPS [85]. Recently, the administration of LPS through the nasal route in mice has gained popularity. A chronic administration via the nasal cavity leads to an approximate loss of 50% of DA neurons from the SNpc and reduces the amount of striatal DA within a month. This phenotype is accompanied by microglial activation, motor deficits, and a twofold increase of expression of α-syn in the SNpc [80, 90]. Lastly, bilateral administration can also be performed to increase the severity of the phenotype [91]. This route of administration could be used to better understand the involvement of environmental exposure through the nasal cavity in developing PD. Overall, LPS-induced PD models are useful in studying the role of neuroinflammation in PD (Table 1). However, the LPS-induced models could involve a more extensive phenotype that is less specific to PD characteristics. In fact, systemic LPS can be used as a chronic inflammation model of Alzheimer’s disease since it can cause memory deficits and an increase in Tau phosphorylation [92–95].

## Agrochemical-induced models

Pesticide exposure is highly associated with PD development and is believed to influence its onset significantly. Epidemiological studies found that working in farming fields and pesticide exposure are positively associated with an increased risk of developing PD [96–98]. Rotenone, paraquat, and maneb are three pesticides that have been linked with PD onset [99, 100].

### Rotenone

Rotenone was first introduced as a PD model in 2000 [101]. After systemic administration, the lipophilic property of rotenone allows it to cross the BBB and diffuse across the cellular membranes of neurons, inhibiting MCI and proteasomal activity. It induces oxidative stress and α-syn accumulation [102, 103]. In rodents, selective degeneration of the DA neurons from the SNpc and axonal denervation in the striatum arise within weeks, accompanied by motor impairments and α-syn inclusions [101, 104, 105]. Consistent with what is observed in idiopathic PD, rotenone activates microglia and impairs autophagy [106–108]. In lower-order species, such as *C. elegans* and *Drosophila*, rotenone can be used to mimic PD by inducing motor deficits and a loss of DA neurons [109]. Due to the genetic simplicity of these species, fast screening approaches can be used when looking for novel neuroprotective targets (Table 1). While rotenone reproduces several features of PD, the main limitation remains in its significant inter-individual variability [110, 111]. This might explain why no studies are reported using rotenone in non-human primates since a greater sample size would be required to account for variability [112].

### Paraquat and Maneb

Paraquat is one of the most widely used herbicides in the world [113]. The mechanism of paraquat leading to PD remains unclear because it cannot cross the BBB. DAT does not directly transport paraquat, but paraquat can be converted to PQ2+, which seems to be the active agent penetrating the DA neurons through DAT [114] (Fig. 2). In rodents, paraquat administered alone has a mild effect on nigral neurons. This herbicide induces partial degeneration of DA cell bodies in the SNpc, without clear denervation of striatal fibers or motor impairments [115–118]. It also transiently increases the amount of α-syn protein leading to inclusions within SNpc DA neurons [119, 120]. Additionally, paraquat drives neuroinflammation and microglial activation [121]. However, particular attention must be given to the paraquat dose since it can result in pulmonary fibrosis, which might interfere with the motor behavioral assessment [122].

In agriculture, paraquat is often used in combination with other agrochemicals, such as maneb. The combined administration of paraquat and maneb in rodents increases the toxicity by inducing microglial activation, a significant loss of DA neurons from the SNpc and striatal fibers, as well as motor impairments [115, 117, 118, 123, 124]. However, in rats, the combined exposure does not seem to affect striatal DA fibers [115, 123]. Over the last two decades, interest has grown in using paraquat to mimic PD in animal models such as *Drosophila* [125] and *C. elegans* [126] since it efficiently causes the loss of DA neurons, and increases ROS levels, cellular dysfunction and motor impairments. In these species, this agrochemical can be used to perform neuroprotective studies by fast screening or to study the gene interaction pathogenesis (Table 1). Although rats are an exception, paraquat and maneb models successfully reproduce many PD hallmarks.

## PD-associated gene models

It is estimated that up to 10% of diagnosed cases of PD have a familial origin [2]. Several genes have been identified as risk factors for PD, including Parkin RBR E3 ubiquitin-protein ligase (*PRKN*), PTEN-induced putative kinase 1 (*PINK1*), Leucine-rich repeat kinas 2 (*LRRK2*), Daisuke-Junko-1 (*DJ-1*), ubiquitin carboxy-terminal hydrolase L1 (*UCH-L1*), and β-glucocerebrosidase (*GBA1*) [127]. In animals, the manipulation of most of these genes can mimic some features of idiopathic PD, such as Lewy body-like formation, and lysosomal and mitochondrial dysfunction. The *SNCA* gene, encoding α-syn protein, is also heavily implicated in genetic and sporadic forms of PD [128]. Existing *SNCA* transgenic models will be discussed in the following section.

### PRKN and PINK1

Of all the familial forms of PD, *PRKN* is the most frequently mutated gene accounting for about 50% of early-onset genetic cases, followed by mutations in *PINK1*, which represent up to 8% of early-onset familial forms of PD [127]. *PRKN* and *PINK1* are both involved in the mitochondrial stress response and are believed to act along the same pathway. Indeed, mitochondrial stress leads to the accumulation of PINK1 within mitochondria and initiates autophagy of damaged mitochondria through the recruitment of PRKN [129] (Fig. 2). *PRKN* and *PINK1* mutations are recessive and generally lead to a loss of function. Interestingly, almost all clinical cases of *PRKN*-induced parkinsonism lack Lewy body pathology [130].

In *C. elegans* and *Drosophila*, genetic deletion of the ortholog form of human *PRKN* and *PINK1* results in typical Parkinson-like phenotypes. In *C. elegans*, these mutations are associated with motor impairment, mitochondrial dysfunction, and loss of DA neurons [131, 132]. In *Drosophila*, *PINK1* ortholog deletions lead to the development of similar features, with the addition of the degeneration of muscles [133]. *PRKN* ortholog-deficient *Drosophila* have a comparable phenotype, except for the absence of DA neuron loss [134]. These models are particularly suitable for fast screening approaches when studying PRKN/PINK1 regulatory mechanisms involved in the pathogenicity. In mice, the genetic deletion of both *PINK1* and *PRKN* leads to mitochondrial dysfunction and neuroinflammation without affecting the basal mitophagy. This model does not appear to exhibit α-syn aggregates, behavioral abnormalities, reduced striatal DA levels, or loss of nigral DA neurons [135, 136]. However, it is possible that neurodegeneration only occurs in older mice. In fact, a recent study showed that hind limb defects and loss of DA neurons in the SNpc can be seen in 2-year-old *PRKN* knock-out (KO) mice [137]. Thus, mice lacking PINK1 or PRKN might be used to better understand the role and the involvement of these genes *in vivo*. Nevertheless, the time required for these mice to develop neurodegeneration could be a major limiting factor for using this model in neuroprotective studies. Recently, a non-human primate model with a genetic deletion of *PINK1* was generated. This model presents behavioral abnormalities at 1.5 years of age and DA neuron degeneration in the SNpc at 3 years of age, without changes in mitochondrial morphology [138]. It is still unknown if these animals develop Lewy body-like inclusions. While the loss of *PINK1* more closely reflects parkinsonian phenotypes, this model is not yet fully characterized. Further studies are needed to assess its importance as a PD animal model (Table 1). Although promising and relevant to PD, no *PRKN*-transgenic model in non-human primates has been generated. This model requires specialized expertise and installations to maintain these animals, which may exceed the technical capacities of many research teams.

### LRRK2

Mutations in the *LRRK2* gene are dominant and account for around 4% of familial cases of PD [139]. LRRK2 is a protein containing both a kinase and a GTPase domain. In addition, this gene interacts with many proteins, regulating various cellular functions (Fig. 2), including autophagy, mitochondrial functions, and vesicular trafficking [140]. Most *LRRK2* mutations, such as G2019S and R1441C/G, affect one of the enzymatic domains. Hyperactivity of LRRK2 kinase has also been observed in idiopathic cases [141]. While DA neuron loss from the SNpc is one characteristic of parkinsonian patients carrying a *LRRK2* mutation, approximately 21%–54% of these clinical cases do not show apparent Lewy bodies [149, 150], suggesting that this mutation could induce neurodegeneration through another factor than synucleinopathy. Some evidence indicates that the toxic effect might be mediated through the protein Tau, since about 79% of PD patients carrying a *LRRK2* mutation show some features of Tau pathology [142]. In *C. elegans* and *Drosophila*, overexpression of human wild-type (WT) or G2019S and mutated *LRRK2* causes motor dysfunctions and progressive degeneration of DA neurons [144–148]. *LRRK2* G2019S overexpression in both species results in mitochondrial and autophagic dysfunction [144, 146, 147, 149]. In *Drosophila*, overexpression of the G2019S mutated form of LRRK2 induces a loss of dendritic arborization by hyperphosphorylation of endogenous Tau. Furthermore, overexpression of the *Drosophila* ortholog of WT LRRK2, Lrrk, has been seen to increase the neurotoxicity of pathological Tau when the human mutated R406W form of Tau is expressed [148]. The *Drosophila* model can therefore be useful in understanding the interaction of LRRK2 and Tau pathology. Moreover, as *C. elegans* and *Drosophila* do not naturally express α-syn, the function of LRRK2 can additionally be studied in the absence of endogenous α-syn.

In mice, G2019S *LRRK2* overexpression and Knock-In (KI) models generate synaptic alterations, impaired DA transmission, behavioral abnormalities and increased Tau phosphorylation [150–156]. These rodent models usually lack degeneration of DA neurons in the SNpc, unless the mouse line overexpresses G2019S *LRRK2* under the neuron-specific platelet-derived growth factor beta (PDGF-β) promoter. These transgenic mice show striatal denervation, DA neuron degeneration, and increased phosphorylated Tau protein in the SNpc at 16 months of age [157]. Features of neuroinflammation, as well as autophagic and mitochondrial abnormalities are also present despite the absence of synucleinopathies [158, 159]. More recently, neurodegeneration has been reported in a mouse line expressing G2019S *LRRK2* in DA neurons under the TH promoter. The alteration drives a progressive loss of DA neurons in the SNpc by 15 months of age, with additional neuronal loss observed in the olfactory bulb and locus coeruleus. By 24 months of age, this phenotype is accompanied by reduced synaptic vesicles in the DA neurons, motor deficits, and an increased amount of phosphorylated α-syn [160] (Table 1). Overall, *LRRK2* mutant mice might be used to study the pathogenesis of PD and to identify neuroprotective therapies aimed at inhibiting its kinase activity or its downstream targets.

### DJ-1

First discovered as a novel oncogene in 1997 [161], the *DJ-1* gene encodes a protein of the peptidase C56 family. It is also known for its function against oxidative stress [162, 163] via its direct interaction with mitochondria [164] and DA neurons in the SNpc, which drive the nigrostriatal motor pathway inhibition (Fig. 2) [165]. Deficiency of DJ-1 has been identified as a causative factor in familial PD with recessive inheritance [166], where it downregulates the level of lysosomal 70 kDa heat-shock cognate protein. By simultaneously inhibiting the activation of chaperone-mediated autophagy, DJ-1 deficiency may augment α-syn aggregation [167] and mitochondrial abnormalities [168] in PD. Although *DJ-1* KO mice exhibit alterations in DA metabolism, the KO alone is not sufficient to induce loss of DA neurons and PD-like signs in young mice. However, in older mice, there are significant DA neuronal losses and motor deficits [169]. A similar age-dependent difference is also visible in the *Drosophila* model with two existing orthologs for DJ-1 [170]. While the major limitation of this model lies within the prolonged time required for a phenotype to appear, it remains promising, as ageing seems to be a crucial factor in progressive neurodegenerative diseases like Parkinson’s. Because PD similarly evolves in older adults, this progressively developing model may be suitable for targeting the earlier phases of the phenotype induction and its subsequent manifestation.

Recent studies have used MPTP hybrid models that take advantage of the usual role DJ-1 has as a negative regulator of the inflammatory response, alleviating neuroinflammation, and playing an essential role in neurodegeneration [171]. *DJ-1* KO mice tend to present PD-like pathology after MPTP treatment, resulting in accelerated DA axonal denervation in the striatum, DA neuron loss in the SNpc, and motor impairments after 2 weeks [165]. The MPTP treatment accelerates the phenotype seen in KO models alone by increasing the oxidative stress burden [163] (Table 1).

### UCH-L1

UCH-L1 is a deubiquitinating enzyme believed to be one of the most abundant proteins in neurons, accounting for about 1%–5% of the total amount of proteins in the brain [172]. The first association between the *UCH-L1* gene and PD was revealed in 1998, following the identification of the autosomal mutation I93M in the pedigree of a German family [173]. Although this mutation is rare, UCH-L1 localizes with Lewy bodies in some sporadic cases of PD [174, 175]. While UCH-L1 is involved in the clearance of proteins via the ubiquitin-proteasome system, a recent study also suggests that UCH-L1 can modulate mitochondrial functions by regulating the expression of Mitofusin-2 [176] (Fig. 2). To date, there are relatively few transgenic animal models for UCH-L1. In mice, the deletion of UCH-L1 induces axonal degeneration in sensory and motor nerve terminals [177–179] without affecting DA nigral neurons [175]. The only transgenic mouse model that affects the DA neurons of the SNpc is the UCH-L1 I93M mice, which overexpress I93M mutated UCH–L1 under the control of the PDGF-β neuron-specific promoter [180]. Although these mice may be useful for studies aimed at discovering neuroprotective therapies for PD by targeting UCH-L1 activity, this approach is limited because *UCH-L1* I93M transgenic mice lack α-syn inclusions. Furthermore, the degeneration of DA neurons and motor deficits can take more than 20 months to develop [180]. To accelerate the degeneration of SNpc DA neurons, this model has been previously combined with viral α-syn overexpression [175]. More recently, knock-down (KD) of the *UCH-L1* ortholog gene (*dUCH*) in a *Drosophila* PD model was created using the GAL4-UAS system. This model exhibits motor alterations, degeneration of DA neurons, and a reduction in the level of DA in the brain [181], thus making this model appropriate for fast screening approaches to identify UCH-L1 interactors (Table 1).

### GBA1

Mutations in *GBA1* are among the most common genetic risk factors for PD and Lewy body dementia [182, 183]. The *GBA1* gene is located on chromosome 1q21 and encodes the lysosomal glucocerebrosidase enzyme (GCase) [184]. Thus, *GBA1* mutations induce a deficiency of GCase activity that leads to an accumulation of glycosphingolipids [185]. Clinical, epidemiological, and experimental studies have confirmed a connection between PD and Gaucher disease, a prevailing autosomal recessive lysosomal storage disorder [186]. Recently, GBA1-derived PD clinical cases have shown impaired DA neurotransmission early in the pathogenesis, although the underlying mechanisms are still not well understood. So far, approximately 130 different *GBA1* mutations have been observed in patients with PD. The most frequent cases with declined GCase activity are the L444P and N370S *GBA1* mutations [185, 187, 188]. Recent studies have focused on the association between mutated *GBA1* and PD, highlighting the possibility that lysosomal impairment and altered GCase activity could facilitate the proliferation of α-syn aggregates [188–190]. Today, several animal models of *GBA1* have been created [182]. *GBA1*-associated PD mouse models combine a *GBA1* mutation with overexpression of α-syn mutations to generate α-syn aggregation and pronounced PD-like signs. Additionally, different KI models carry the human L444P *GBA1* gene [191]. *GBA1* L444P homozygous mice show an increase of α- syn accumulation in the striatum and astrogliosis at 1 year of age [192]. *GBA1* L444P heterozygous mice develop mitochondrial dysfunction, increased ROS production, impaired neuronal autophagic degradation [193], and higher levels of α-syn [194], but fail to develop α-syn aggregates even by 24 months of age [194]. Unlike L444P, heterozygosity for N370S does not affect phenotype progression in A30P-*SNCA* mice [193], and N370S homozygote mice die at birth. Injection of adeno-associated virus (AAV) expressing N370S *GBA1* in the striatum of A53T-*SNCA* mice results in increased α-syn protein, defects in mitophagy, and abnormal autophagy [191, 193]. *GBA1*-associated PD models can also be found in *Drosophila* and *C. elegans*. In these PD-*GBA* *Drosophila* models, an increased aggregation of mutant α-syn A53T is observed, causing enhanced DA neuronal loss and exacerbating the motor and non-motor phenotypes [195]. *Drosophila* expressing the mutant human N370S and L444P variants exhibit a significant decrease in GCase activity and present parkinsonian features with DA cell death, defective locomotion, and a shorter lifespan [196]. Overall, fly models expressing human WT or N370S and L444P *GBA1* provide an interesting tool to assess the contribution of defective GCase function to PD development. In *C. elegans* expressing a human α-syn, KD of GCase ortholog, C33C12.8, increases aggregation of α-syn [197], similar to what has been described in other animal models [198]. In sum, *GBA1*-associated models of PD provide insight into the pathogenesis by replicating many hallmarks of the disease. *GBA1* models show increased α-syn accumulation, disruption of lysosomal homeostasis, exacerbated endoplasmic reticulum stress, and mitochondrial dysfunction. These cellular features lead to DA neuron death and motor phenotypes, making them an appealing tool for studying PD (Table 1).

### Transcription factors

During the development of midbrain DA neurons, transcription factors play key roles in specifying and differentiating neural progenitors. Interestingly, most of these transcription factors remain expressed in the adult brain [199, 200]. Indeed, Lmx1a/b, Otx2, Foxa1/2, Pitx3, Nurr1, and En1/2 are present throughout development and are required for the survival of midbrain DA neurons [201–204]. Genome-wide association studies have revealed genes that may contribute to PD [205]. Among them, genetic variants of human transcription factors such as Lmx1a and Lmx1b were found [204, 206–208], with the expression of Lmx1b being notably decreased in the DA neurons of PD patients [209]. Lmx1a and Lmx1b are LIM homeodomain transcription factors essential for the development of midbrain DA neurons [210–213] and are still expressed in post-mitotic midbrain DA neurons [214–216]. Conditional deletion of *Lmx1a* and *Lmx1b* genes in mouse DA neurons induces progressive neurodegeneration in the SNpc and VTA [203, 217]. Lmx1a and Lmx1b are particularly important in regulating mitochondrial [217] and autophagic-lysosomal functions [209]. Moreover, loss of Lmx1a and Lmx1b leads to α-syn pathology with the accumulation of α-syn in both DA axons and cell bodies [217]. Specific deletion of *Lmx1a* and *Lmx1b* in adult DA neurons also leads to progressive loss of DA neurons, α-syn pathology, and motor impairments [209, 217]. Depletion of Otx2, Foxa1/2, and Pitx3 leads to a reduction of midbrain DA neurons with motor deficits [218–223]. While heterozygous KO mice for *Nurr1* and *En1* develop progressive loss of DA neurons with motor deficits [224, 225], total depletion of Nurr1 [224, 226] and En1 [227] in midbrain DA neurons is embryonically lethal. Lastly, KO of the transcription factor c-Rel can induce α-syn aggregation in the SNpc, midbrain DA neuronal loss, and motor deficits [228] (Table 1). Overall, several signs of PD can be reproduced by modulating the expression of DA transcription factors; however, the phenotype is restricted to DA neurons. Most of these models also require multiple crosses to obtain a conditional deletion in adult mice, which could thus represent a limiting factor.

### Mitochondrial impairment

Several lines of evidence have previously linked mitochondrial dysfunction to PD pathogenesis [229, 230]. This is supported by a large set of PD-associated genes that are involved in mitochondrial function [231, 232]. Furthermore, α-syn and neurotoxins linked to PD, including MPTP and rotenone, are known to inhibit MCI activity [233]. MCI deficiency was also reported in DA neurons of the SNpc for human PD post-mortem brains [234]. One of the first transgenic mouse models of PD to induce mitochondrial impairment in DA neurons was created by crossing DJ-1-deficient mice with a mouse strain encoding a mutated mitochondrial polymerase gamma, resulting in an accelerated accumulation of mitochondrial DNA errors. Even at 1 year of age, these mice do not exhibit behavioral abnormalities, degeneration of nigrostriatal DA neurons, or evidence of neuroinflammation [235]. A similar phenotype has recently been observed in mice lacking PRKN [236].

In mice, conditional deletion of the *Ndufs4* gene (which encodes a subunit of MCI) in DA neurons results in loss of striatal DA at 9 months, followed by increased α-syn phosphorylation in DA neurons at 24 months of age. However, deletion of *Ndufs4* in DA neurons does not cause motor deficits or DA neuron degeneration in the SNpc [237]. One possible explanation for this phenotype is that *Ndufs4* KO results in about 65% reduction of MCI activity [237] and DA neurons could compensate for the lack of oxidative phosphorylation [238]. Recently, another model of MCI disruption by deletion of the essential MCI subunit Ndufs2 in DA neurons was generated. Compared to the *Ndufs4* KO model, the KO of *Ndufs2* almost completely disrupts the oxidative phosphorylation activity in DA neurons. Deletion of *Ndufs2* induces loss of DA axons in the dorsal striatum by 1 month of age, followed by motor deficits after 1 to 2 months. These motor deficits are accompanied by a decrease in the number of cells expressing TH in the SNpc. However, despite the decrease in TH expression, there is no loss of dopaminergic cell bodies [238]. No α-syn aggregates have been observed in this model, but it might be because that these mice die at 5–6 months of age before developing α-synucleinopathies [238]. Additional characterization of neuroinflammatory and autophagic features is still needed. However, the *Ndufs2* KO mouse model remains of interest in the study of neuroprotective therapies aiming to rescue oxidative phosphorylation or MCI activity.

## α-syn models

The α-syn protein, encoded by *SNCA*, has a central role in PD, although its physiological function is not yet fully understood. Usually clustering into monomers and tetramers [239, 240], α-syn assembles into higher-order structures under pathological conditions. These oligomers, protofibrils, and fibrils form insoluble aggregates that are toxic to neurons [241]. As a principal constituent of Lewy bodies [242], these aggregates can not only alter the integrity of the cell membrane, but may also disrupt the functions of mitochondria, the Golgi apparatus, and the endoplasmic reticulum [243] (Fig. 2). At least eight different identified missense mutations of *SNCA* (A53T/E/V, A30P/G, E46K, H50Q and G51D) [244], gene duplication, and triplication have been linked to the accumulation of pathologic α-syn in the brain [245]. This new knowledge gave rise to several animal models mimicking α-syn pathology. Transgenic models expressing WT, A53T, A30P, and E46K *SNCA* mutations have been utilized to recapitulate PD pathology *in vivo*. However, to date, there are no reported studies on A53E, A53V, A30G, H50Q, or G51D transgenic models, which could be of interest for future investigations.

### WT and truncated human α-syn

As one of the first transgenic animal models of α-syn, the mouse line 61 (M61) is generated by overexpressing human WT α-syn using the neuron-specific Thymocyte differentiation antigen 1 (Thy1) promoter [246]. Mitochondrial dysfunction and neuroinflammation have been described in these mice [247, 248]. Moreover, motor deficits and α-syn inclusions appear within a few months of age, although nigral DA neurons are only mildly affected. In fact, while a decreased amount of striatal DA and axonal denervation are observed, no degeneration of DA neurons in the SNpc is reported [246, 249]. M61 mice have thus been used to test candidate drugs targeting α-syn aggregation and axonal denervation. The MI2 mouse line expressing C-terminal-truncated human α-syn under the TH promoter shows α-syn aggregates in DA neurons by 1.5 months of age. By 9 months, motor impairments appear; by 12 months, a significant loss of DA neurons in the SNpc can be seen [250]. Additionally, because MI2 mice possess a null endogenous α-syn background, human α-syn seeding is facilitated. However, since the α-syn expression is restricted to DA neurons, the MI2 mouse line does not recapitulate synucleinopathies in other brain regions.

### E46K human α-syn

Identified within a Spanish family with autosomal dominant parkinsonism and dementia, the cortical and subcortical Lewy body pathology found was attributed to a shared nonconservative heterozygous E46K mutation within α-syn [251]. While the mutant mouse PD models created based on this mutation are still recent and warrant further research, this initiative can provide insight into familial PD and synucleinopathies. Transgenic mouse lines carrying the PrP (Prion protein)-driven E46K mutation have been generated. The mouse line 47 (M47) shows higher levels of α-syn transgene expression, with motor impairments starting at 15–16 months of age. These mice develop α-syn inclusions in the spinal cord, brainstem, deep cerebellar nuclei, motor cortex, and most of the thalamus. The inclusions and affected areas are accompanied by phosphorylation of α-syn, significant astrocytic gliosis, elevated reactive microglia, and Tau inclusions [252]. DA neurons of the SNpc remain spared of α-syn inclusions. These results may corroborate those observed in rat models with the same mutation, where no loss of DA neurons or striatal terminals was reported [253].

### A30P α-syn

The A30P α-syn mutation was first detected in a German family [254]. The few existing transgenic mouse strains that model the A30P mutation include overexpression of the human A30P α-syn gene under the Prp promoter (Prnp-A30P) [255] or the Thy1 promoter (Thy1-A30P) [256], and a KI mouse model with an introduced A30P point mutation in the WT gene [255]. All these A30P models show an increase and accumulation of α-syn in neuronal cell bodies with age. However, it is most robust in the Prnp-A30P mice. For Thy1-A30P α-syn transgenic mice, abnormal α-syn protein is observed in several brain regions like the superior colliculus or the cerebellum, but α-syn pathology is spared in the striatum and SNpc [255]. Mitochondrial oxidative stress and autophagy defects are also reported in A30P models [257]. Only the neuronal cell loss in the SNpc does not seem to be consistently detectable. While Thy1-A30P mice show reduced TH levels in the SNpc at 8 and 11 months [256], the loss of DA is not visible in the strains [255, 258]. Locomotor disabilities and α-syn aggregation seen in rodent models are similarly observed in flies overexpressing the A30P mutant form of α-syn. Flies also exhibit selective loss of DA neurons when A30P is expressed in all neurons [259, 260]. In *C. elegans*, A30P α-syn models are mild. Compared to control animals, worms expressing A30P α-syn in muscles have modest reduction of movement speed and increased rate of paralysis [261]. In a *C. elegans* model of A30P under the control of the DAT-1 (*C. elegans* DAT) promoter, the expression is restricted to DA neurons. The worms exhibit DA neuron-specific dysfunction caused by accumulation of α-syn but do not show significant neurodegeneration compared to controls, even at 15 days of age [262]. Overall, the A30P models show mild phenotypes. The progressive and relatively long development of pathological abnormalities, such as α-syn accumulation and motor disorders, may provide an interesting approach to study the early stages of PD.

### A53T human α-syn

The A53T mutation in the α-syn gene was initially identified in Italian and Greek pedigrees [263]. The PrP-driven mouse line 83 (M83) was the first to be genetically engineered to overexpress human A53T α-syn. M83 mice show a mid-to-late onset of neurodegenerative phenotype with evidence of dystrophic neurites. They present increased phosphorylated α-syn and aggregation, similar to patients [264–267]. These inclusions become apparent between 8 to 16 months of age in homozygous M83 mice, resulting in motor impairments. Like M47 mice, the highest density of these inclusions is localized in the spinal cord, brain stem, deep cerebellar nuclei, white matter, and some thalamic regions. An identical inclusion profile arises in hemizygous M83 mice between 22 and 28 months of age when animals spontaneously develop motor dysfunction [264]. No loss of DA neurons has been reported, and there is no alteration of striatal DA at 12 months of age in M83 homozygous mice [268]. Altogether, this mutation owes its pathological nature to its tendency to form α-syn inclusions, much like in the clinical setting. While the location of pathological lesions in mice does not precisely mirror the human condition, they do share many similarities, such as neuroinflammation, as well as autophagic and mitochondrial dysfunction [269–272]. This human A53T mutation model is proposed to be a valuable asset in screening potential therapeutics that inhibit or reverse α-syn aggregate formation or spreading.

### α-syn preformed fibrils (PFFs)

PFFs are first generated from monomeric recombinant α-syn. When fragmented by sonication, PFFs can phosphorylate endogenous α-syn into its pathological state [273]. In mice, α-syn spreading is enhanced with PFFs generated from brain homogenates of aged symptomatic α-syn mice or human samples, or recombinant synthetic α-syn fibrils (Fig. 2). In WT mice, injection of mouse PFFs in the dorsal striatum induces hyperphosphorylation of α-syn within 30 days [274]. Degeneration of DA striatal fibers and cell bodies in the SNpc, as well as motor impairments, occurs 6 months post-injection [274]. Whole-brain homogenate extracts of parkinsonian symptomatic M83 mice can be injected into the M83 mice at birth to accelerate the development of the phenotype and α-syn aggregation [275–277]. There is also a robust inflammatory response before end-stage paralysis occurs by 8 to 16 months in homozygous mice and by 22 to 28 months in heterozygous mice [278]. Similarly, extracts from post-mortem PD brain tissues have previously been used in the intranigral and intrastriatal inoculation of WT mice and macaque monkeys. The fractions enriched with nigral Lewy bodies have demonstrated the ability to induce pathogenic effects, such as progressive nigrostriatal neurodegeneration and the accumulation of pathological α-syn, which cannot be achieved by non-Lewy body fractions or vehicle buffers [279]. A new alternative created with the bacterial artificial chromosome plasmid consists of A53T overexpressing mice injected with recombinant synthetic α-syn. This hybrid model produces phosphorylated α-syn inclusions and striatal DA denervation within 2 weeks post-injection [280]. Within 1 month, there is 25% DA neuronal loss in the SNpc. Motor impairments were reported at 2 months post-injection [280], but they can be expected to appear earlier since DA denervation appears before this time point [277]. In cell-based models, PFFs were shown to additionally produce autophagic and mitochondrial dysfunction [281, 282]. Lastly, recent studies in 26-month-old non-human primates have shown that PFFs are also sufficient to drive phosphorylated α-syn accumulation and DA neuronal loss by 3 months post-injection [273]. However, the injection site of the PFFs is consequential in further characterizing the α-syn seeding and spreading [274, 277, 283]. We cannot overlook that α-syn transmission also occurs in the periphery and that α-synucleinopathy in the brain may develop via the gut-to-brain axis [284]. Inoculation of the rodent gut with PFFs results in midbrain DA neuron loss, α-syn histopathology, and motor defects in a temporal manner [285–287]. Nevertheless, despite differences from the human pathology, PFFs can reproduce specific aspects or stages of α-syn pathology, as well as neurodegenerative and neuroinflammatory hallmarks of PD [288]. Overall, both *in vitro* and *in vivo*, fibril formation and aggregation are not synonymous with pathological aggregation and LB formation. Therefore, strict quality control must be taken so that the pathogenicity of the PFFs used ensures reproducible results [284, 289, 290].

### Non-mammalian models of α-syn

Non-mammalian models, including those based on *C. elegans* and *D. melanogaster*, do not produce α-syn endogenously. In *C. elegans* [291–293] and *Drosophila* [294–296], the overexpression of human WT and A53T α-syn in an ubiquitous or DA-specific manner induces α-syn aggregates, mitochondrial fragmentation, motor impairments, and loss of DA neurons. These non-mammalian models offer an interesting way for rapid screening of anti-aggregation targets. However, they might be too simplistic to transpose the results directly to complex and still not fully understood mechanisms of aggregation, propagation, and toxicity of synucleinopathies in the human brain.

## Viral vector-mediated models

### α-syn viral expression

Another alternative to model PD in animals is to overexpress WT or mutated α-syn, including A53T and A30P forms [297], using lentiviral [298] or AAV vectors [299, 300]. These viral vectors can transduce diverse cell types and drive the expression of α-syn in the brain region of interest [301]. This technique thus makes it feasible to study the possible transmission of α-syn pathology from the periphery to the central nervous system [302]. In general, α-syn overexpression in the SNpc results in the progressive DA neuron loss, and DA projection decrease in the striatum within weeks to months, and models early and late hallmarks of PD pathogenesis, depending on the model organism [300, 303–305]. However, studies have yielded variable results, showing a range from slight to substantial loss of DA neurons. Several factors, including virus titer or serotype, age, sex, strain, or species of the animal, can strongly influence the outcomes obtained (see [306, 307] for reviews). In rodents, viral α-syn overexpression results in an approximate 40% DA cell loss in the SNpc, accompanied by motor deficits at 8 weeks post-injection [308]. Less commonly used, non-human primates receiving intranigral AAV injections of WT or A53T α-syn show a ~ 30% loss of nigral DA neurons 8 weeks post-injection. Like rodents, non-human primates also develop motor deficits with a progressive deterioration of motor coordination after 16 weeks, albeit with a greater variability [309]. Non-human primates enable a more extended period of studies on the effects of α-syn injections than their rodent counterparts [310, 311]. Moreover, monkeys were found to be more vulnerable to neurodegeneration and pathologies induced by injections with viral vectors that encode A53T α-syn [311]. Overexpression of α-syn in the midbrain typically leads to mitochondrial dysfunction [312], impaired autophagy [313], Lewy body-like structures [300, 314], and an early and persistent inflammatory response [315–318]. Recently, a light-inducible α-syn aggregation system (LIPA) was designed to regulate spatiotemporal protein aggregation [319]. This system could control α-syn aggregation into insoluble deposits that mimic key features of Lewy bodies. LIPA is encoded in a viral vector and injected into the SNpc. The α-syn aggregation is then induced by light stimulation through an optical fiber implanted above the injection site. Following repeated optogenetic stimulations, the nigrostriatal transmission is compromised, leading to Lewy body-like structure formation, neurodegeneration, and motor impairments within 2 months. This inducible model of α-syn aggregation allows for real-time monitoring of α-syn aggregation and Lewy body-like formation with high spatiotemporal control. Altogether, viral vectors are very versatile and powerful tools that offer the opportunity to closely mimic PD pathology by inducing the overexpression of α-syn within multiple animal species (Table 1). Viral overexpression allows us to study α-synucleinopathy more precisely and to follow the progressive development of PD-like phenotypes. Lastly, this tool may also target other non-dopaminergic systems, following the impact of α-syn in the periphery or other neuronal cell populations.

### Viral neuromelanin (NM)-like production

Neurodegeneration is particularly prominent within brain regions containing NM. In 1919, a visually noticeable loss of pigmented neurons in the SNpc was first reported by Konstantin Tretiakoff [320]. This pigmentation loss in the brains of PD patients referred to a decrease in NM concentration, which was less than 50% of the expected levels for age-matched healthy individuals [321]. NM pigment appears black and consists of melanin, proteins, lipids, and metal ions. It can be found in different human central nervous system neurons and is particularly abundant in DA neurons of the SNpc and noradrenergic neurons of the locus coeruleus. The pigment itself is contained within specific types of lysosomes fused with autophagic vacuoles [322]. Additionally, DA and norepinephrine appear to be the principal substrates in its synthesis. However, NM may have both a protective and a toxic effect, depending on the conditions of the neuronal environment. Under physiological conditions, NM is proposed to have a protective role via iron binding. In PD, iron-bound NM can easily release excess iron, producing a toxic effect via redox processes. Moreover, the iron overload of NM can catalyze DA oxidation, forming DA-o-quinone, and modifying proteins that trigger neurotoxic effects [323] (Fig. 2). Yet, the structure and function of human NM in PD have proven to be difficult to investigate due to the limited quantities available for extraction from human brain tissues. Furthermore, animal models including rodents lack NM. Nevertheless, recent *in vivo* models have been explored, reporting microglial activation via intranigral synthetic NM injections [324–326]. A new approach was applied in rodents to virally express human tyrosinase (hTyr) in the SNpc. This model shows NM-like pigment accumulation in SNpc DA neurons in an age-dependent manner. In fact, overexpression of hTyr recapitulates the principal hallmarks of PD: mice show mitochondrial and autophagic dysfunction, as well as neuroinflammation. They also have impaired DA release, striatal denervation, loss of DA neurons in the SNpc, and motor deficits by 4 months post-injection [327]. This is accompanied by Lewy body-like structures and autophagic dysfunction within NM-like positive neurons [327, 328]. Therefore, this model provides a valuable experimental tool to study the effects of NM production and accumulation on neuronal function and viability (Table 1).

## Discussion

Modelling PD pathogenesis remains a difficult task. Several animal models have been developed to understand the pathogenesis and test new drug candidates against PD. However, PD is a highly heterogeneous disease involving several factors and pathways that may differ among clinical cases. Therefore, none of the existing and future models will replicate the entire spectrum of clinical features listed in PD. In this review, we tried to summarize the most relevant PD animal models, their respective advantages and disadvantages, as well as their ability to reproduce the main hallmarks of PD.

The choice of the animal model must be based on the PD features addressed by the question the experimenter seeks to answer. Neurotoxin-induced animal models remain popular due to their cost-effectiveness in generating a PD-like phenotype in a relatively short time span. Thus, they are commonly used in drug validation for symptomatic treatment of PD or cell replacement therapy. On the other hand, to investigate the function of PD-linked genes in disease development, transgenic models should be considered. Other questions concerning the targeting of α-syn pathology can also be answered in animal models injected with PFFs or viral vectors encoding *SNCA* gene copies. In the absence of a suitable model fully recapitulating the targeted PD features of interest, a possible solution could be to complement the phenotypes by combining some of the listed models.

The selection of animal species is equally as important. Although the translational results to humans are valuable in mammalian models, the use of rodents and primates for drug or gene screening is untenable. *C. elegans* and *Drosophila* could be considered to identify novel neuroprotective targets [293, 329]. However, the simplicity and lack of similarity with humans are limitations. Moreover, research in PD should not be limited to the use of *in vivo* models. Although animals offer a better pre-clinical prediction of a drug effect, *in vitro* models could provide complementary information on the underlying molecular mechanisms [330]. Still, most *in vitro*-based models cannot reproduce the non-cell autonomous impact of the complex cell network in the brain. The near future could shed light on the use of human brain organoids to perform drug or gene screenings, although additional characterization of these emerging models will be required [330, 331].

## Conclusion

Despite efforts devoted to interpreting the pathogenesis of PD, the initial source of neuronal degeneration remains unclear. There is considerable evidence to suggest that α-syn aggregation, neuroinflammation, as well as lysosomal and mitochondrial dysfunction could all play a key role in neurodegeneration [3]. Basic research has yet to reconcile these features, discriminating causes from consequences in the PD pathogenesis. Existing animal models, many of which recapitulate multiple disease features from cellular pathology and pathogenesis to motor and non-motor symptoms, open new possibilities. However, the wide range available can make choosing a model for a given study challenging. This task becomes particularly complex if the study addresses multiple aspects of PD. This review compiles information on the diversity of animal models, and is therefore, a support for researchers to select the optimal animal model for their study. Carefully constructed research models are an essential first step to understand the disease and discover novel therapies, possibly improving the quality of life and the clinical fate of patients.

## Data Availability

Not applicable.

## Abbreviations

6-OHDA: 6-Hydroxydopamine
AAV: Adeno-associated virus
α-syn: α-synuclein
BBB: Blood-brain barrier
DAT: Dopamine transporter
DIP: Drug-induced parkinsonism
DJ-1: Daisuke-Junko-1 gene
dUCH: UCH-L1 ortholog gene
GBA1: β-Glucocerebrosidase
GCase: Glucocerebrosidase enzyme
hTyr: Human tyrosinase
KD: Knock-Down
KI: Knock-In
KO: Knock-Out
L-DOPA: Levodopa or l-3,4-dihydroxyphenylalanine
LIPA: light-inducible α-syn aggregation system
LPS: Lipopolysaccharide
LRRK2: Leucine-rich repeat kinase 2
MCI: Mitochondrial Complex I
MPP+: 1-methyl-4-phenylpyridinium
MPTP: 1-methyl-4-phenyl-1,2,3,6-tetrahydropyridine
NM: Neuromelanin
PD: Parkinson’s disease
PDGF-β: Platelet-derived growth factor beta
PFF: Preformed fibril
PINK1: PTEN-induced putative kinase 1
POLG: polymerase gamma
PRKN: Parkin RBR E3 ubiquitin-protein ligase or PARKIN
Prp: Prion protein
ROS: Reactive oxygen species
SNpc: Substantia nigra pars compacta
Tau: tubulin-associated unit
TH: Tyrosine hydroxylase
Thy1: Thymocyte differentiation antigen 1
UCH-L1: Ubiquitin carboxy-terminal hydrolase L1
VTA: Ventral tegmental area
